# TRIM3 facilitates estrogen signaling and modulates breast cancer cell progression

**DOI:** 10.1186/s12964-022-00861-z

**Published:** 2022-04-07

**Authors:** Ting Zhuang, Beibei Wang, Xiaojing Tan, Le Wu, Xin Li, Zhongbo Li, Yuqing Cai, Rongrong Fan, Xiao Yang, Chenmiao Zhang, Yan Xia, Zhiguo Niu, Bingtian Liu, Qi Cao, Yinlu Ding, Zhipeng Zhou, Qingsong Huang, Huijie Yang

**Affiliations:** 1 Xinxiang Key Laboratory of Tumor Migration, Invasion and Precision Medicine, Henan Key Laboratory of Immunology and Targeted Drugs, School of Laboratory Medicine, Henan Collaborative Innovation Center of Molecular Diagnosis and Laboratory Medicine, Xinxiang Medical University, Xinxiang, Henan Province People’s Republic of China; 2 Department of Oncology, Dong Ying People’ S Hospital, Dongying, Shandong Province People’s Republic of China; 3 College of Informatics, Huazhong Agricultural University, Wuhan, 430070 Hubei Province People’s Republic of China; 4 Department of Bioscience and Nutrition, Karolinska Institute, 14157 Huddinge, Sweden; 5 Department of General Surgery, The Second Hospital, Cheeloo College of Medicine, Shandong University, Jinan, 250033 Shandong Province People’s Republic of China; 6 College of Life Science and Technology, Huazhong Agricultural University, Wuhan, 430070 Hubei Province People’s Republic of China

**Keywords:** TRIM3, ER alpha, Breast cancer, Ubiquitin, Stability

## Abstract

**Background:**

Breast cancer is the most common cancer in women worldwide. More than 70% of breast cancers are estrogen receptor (ER) alpha positive. Compared with ER alpha-negative breast cancer, which is more aggressive and has a shorter survival time, ER alpha-positive breast cancer could benefit from endocrine therapy. Selective estrogen receptor modulators, such as tamoxifen, are widely used in endocrine therapy. Approximately half of ER alpha-positive breast cancer patients will eventually develop endocrine resistance, making it a major clinical challenge in therapy. Thus, decoding the throughput of estrogen signaling, including the control of ER alpha expression and stability, is critical for the improvement of breast cancer therapeutics.

**Methods:**

TRIM3 and ER alpha protein expression levels were measured by western blotting, while the mRNA levels of ER alpha target genes were measured by RT–PCR. A CCK-8 assay was used to measure cell viability. RNA sequencing data were analyzed by Ingenuity Pathway Analysis. Identification of ER alpha signaling activity was accomplished with luciferase assays, RT–PCR and western blotting. Protein stability assays and ubiquitin assays were used to detect ER alpha protein degradation. Ubiquitin-based immunoprecipitation assays were used to detect the specific ubiquitination modification on the ER alpha protein.

**Results:**

In our current study, we found that TRIM3, an E3 ligase, can promote ER alpha signaling activity and breast cancer progression. TRIM3 depletion inhibits breast cancer cell proliferation and migration, while unbiased RNA sequencing data indicated that TRIM3 is required for the activity of estrogen signaling on the -genome-wide scale. The immunoprecipitation assays indicated that TRIM3 associates with ER alpha and promotes its stability, possibly by inducing K63-linked polyubiquitination of ER alpha. In conclusion, our data implicate a nongenomic mechanism by which TRIM3 stabilizes the ER alpha protein to control ER alpha target gene expression linked to breast cancer progression.

**Conclusion:**

Our study provides a novel posttranslational mechanism in estrogen signaling. Modulation of TRIM3 expression or function could be an interesting approach for breast cancer treatment.

**Graphical abstract:**

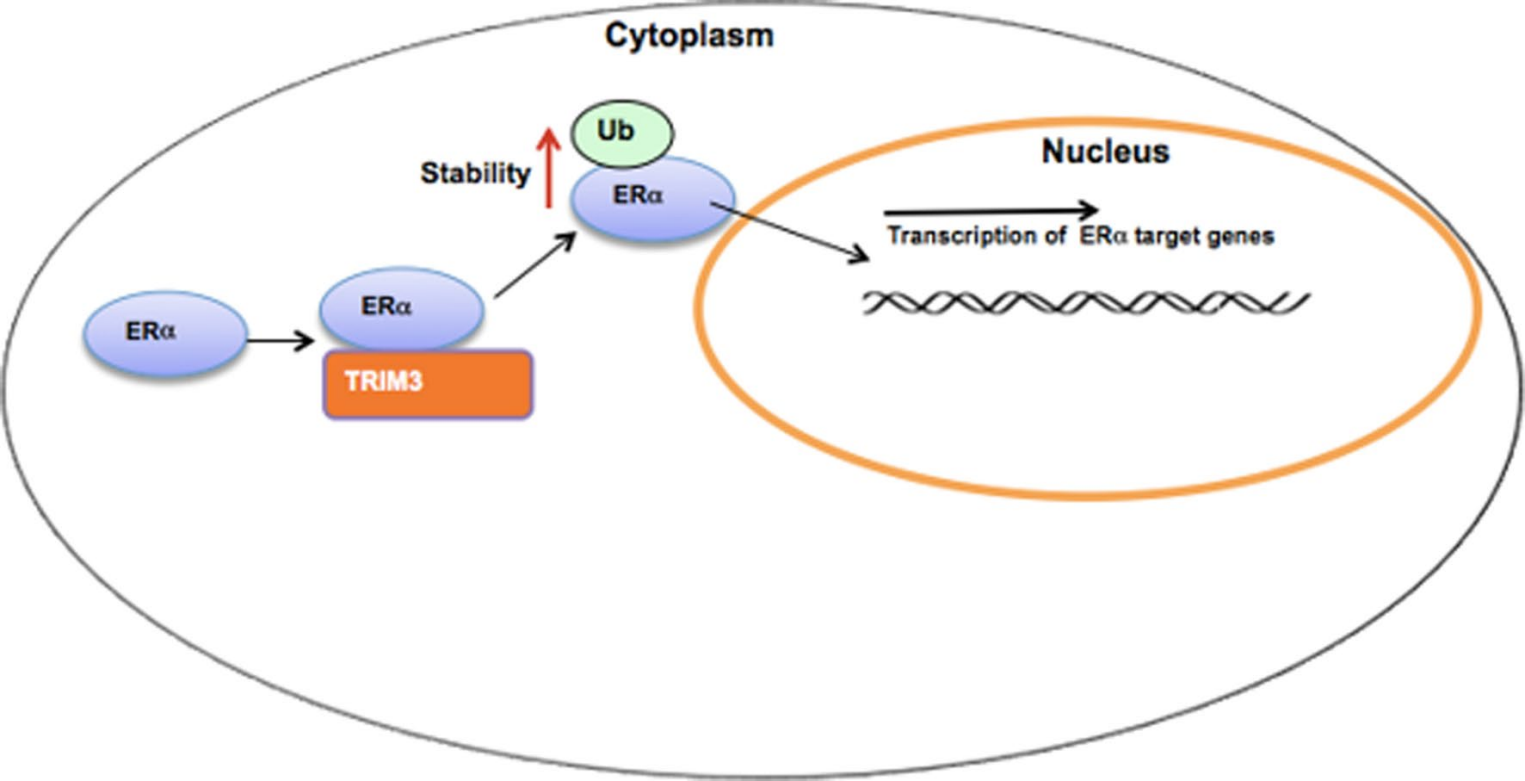

**Video abstract**

**Supplementary Information:**

The online version contains supplementary material available at 10.1186/s12964-022-00861-z.

## Background

Breast cancer is the most prevalent malignancy among women and there are approximately more than 1 million new cases worldwide each year [1]. Genome-based profiling analysis showed that breast cancer can be classified into four molecular subtypes: luminal A, luminal B, HER2 and basal-like [2]. Among the subtypes, luminal A and B account for approximately two-thirds of cases and could benefit from endocrine therapy [3]. Selective estrogen receptor modulators (SERMs) have become the standard treatment for ER alpha-positive patients [4]. However, more than half of patients will develop resistance to endocrine therapy, which is a major challenge in breast cancer treatment [5]. Although ER alpha expression is lost in some breast cancer cells, most endocrine-resistant breast cancer cells still maintain ER alpha expression [6]. Thus, it is necessary for scientists to characterize both the resistance mechanism and the internal mechanism that controls ER alpha expression and stability.

Estrogen receptor (ER) alpha belongs to the superfamily of nuclear receptors and was initially cloned from MCF-7 cells in 1985 [7]. The ER alpha protein is composed of 595 amino acids and contains three functional domains: a DNA binding domain, transcriptional activation domain and ligand binding domain [8]. When ER alpha is activated by 17β-estradiol (E2), the ER alpha protein can translocate into the nucleus and subsequently bind to the promoter regions of ER alpha target genes, which facilitates the expression of certain downstream target genes, such as PS2 and GREB1 [9]. In addition, the effect of ER alpha ligands and the ER alpha protein can be regulated by several posttranslational modifications. These modifications can be widely distributed throughout the ER alpha protein and affect ER alpha function in several ways. For example, acetylation of the ER alpha protein by P300 can enhance ER alpha activity [10], while phosphorylation of the ER alpha protein at Y537 by SRC kinase can change its conformation and increase its ligand-independent activity [11]. Recent studies have revealed that several atypical ubiquitination mechanisms could also play important roles in modulating ER alpha function. For example, our previous studies showed that RNF31/SHARPIN modulates ER alpha monoubiquitination and facilitates ER alpha stability in breast cancer cells [12, 13]. Based on previous studies, we inferred that the components of the ubiquitin–proteasome system, including several E3 ubiquitin ligases, might control ER alpha ubiquitination, turnover, and ER alpha target gene expression.

TRIM3 (tripartite motif containing 3) is a RING finger protein that was initially found to be a regulator of myosin function and to facilitate protein transport in cells [14, 15]. Several studies have shown decreased expression levels of TRIM3 and identified its potential role as a tumor suppressor in quite a few human cancers [16–19]. However, our data showed that TRIM3 was specifically related to poor survival in patients receiving endocrine therapy. Our data demonstrated TRIM3 as a candidate factor in modulating estrogen signaling in breast cancer cells. TRIM3 associates with ER alpha; facilitates ER alpha stability, possibly by inducing ER alpha monoubiquitination; and enhances ER alpha signaling activity. Our study revealed a novel nongenomic regulatory relationship between TRIM3 and ER alpha signaling in breast cancer progression.

## Materials and methods

### Cell culture

MCF-7, T47D and HEK293 cells were acquired from the American Type Culture Collection (ATCC). MCF-7 and HEK293 cells were cultured in Dulbecco’s modified Eagle’s medium supplemented with 4.5 g/L glucose, 4 mM l-glutamine (DMEM, 41965, Life Technologies) and 10% fetal bovine serum (FBS, 10270, Life Technologies). T47D cells were cultured in RPMI-1640 medium (42401, Life Technologies) supplemented with 2 mM l-glutamine (25030, Life Technologies) and 10% FBS. 17b-estradiol (E2; Sigma-Aldrich) was dissolved in ethanol. For adding E2 assays, cells were cultured in charcoal-stripped FBS (Gibco, 12676-029) treated with phenol red-free DMEM (Gibco, 11330-057). All cell lines were characterized by cell line authentication. Cell line authentication via short tandem repeat (STR) was performed via the Promega Power Plex 21 system. The STR data of the MCF-7 and T47D cell lines were consistent with the STR data in ATCC.

### Plasmids and siRNA

The Flag-TRIM-3 plasmid was acquired from OriGene. The ER alpha full-length and deletion constructs were described in a previous study [20]. The TRIM3 and ER alpha deletion variants were subcloned from the original plasmids. The HA-Ub-K48, HA-UbKO, HA-Ub-K63 and HA-Ub plasmids were used in a previous study. The estrogen response element (ERE)-TK reporter and Renilla plasmids were used in a previous study and transfected with Lipofectamine 2000 (1662298, Invitrogen). Small interfering RNAs were used for knockdown of specific genes knocking down. The website of https://www.sigmaaldrich.cn/ was used to design the sequence of siTRIM3. The TRIM3 siRNA sequences were CAAACGAAAGGACAACCCAdTdT, UGGGUUGUCCUUUCGUUUGdTdT, GCAACAACCAGUGUAUUCAdTdT, and UGAAUACACUGGUUGUUGCdTdT. The negative control siRNA sequences were UUCUCCGAACGUGUCACGUTT and ACGUGACACGUUCGGAGAATT. RNAiMAX reagent (13778150, Invitrogen) was used for siRNA transfection.

### RNA extraction and RT–PCR analysis

RNeasy plus mini kits were used to extract total RNA (Qiagen). RT–PCR was performed as previously described [21]. 36B4 was used as the internal control. The primer sequences were as follows. 36B4 F: GGCGACCTGGAAGTCCAACT, R: CCATCAGCACCACAGCCTTC. GREB1 F: CGT GTG GTG ACT GGA GTA GC, R: ACC TCT TCA AAG CGT GTC GT. ER F: GCT ACG AAG TGG GAA TGA TGA AAG, R: TCT GGC GCT TGT GTT TCA AC. PS2 (TFF1) F: TGG GCT TCA TGA GCT CCT TC, R: TTC ATA GTG AGA GAT GGC CGG. The PrimerBank (harvard.edu) was used to design the sequence of primers used.

### Quantification of cell viability

MCF-7 and T47D cells were transfected with siTRIM3 or siControl in 24-well plates. Twenty-four hours after transfection, the cells were counted, and 4000 cells were seeded into 96-well plates. The relative cell viability was measured at the indicated time points. Cell numbers were determined using WST-1 cell proliferation reagent as previously described [21].

### Wound healing assay

Fifty nanograms of TRIM3 siRNA or siControl was transfected into MCF-7 or T47D cells. After 24 h, cells were seeded into 12-well plates in medium with 1% FBS. The cells were 100% confluence. Yellow pipette tips were applied for scratching straight lines. The wound width was measured at the indicated time points and normalized to the width at the initial time point. The wound healing recovery was expressed as [1 − (width of the wound at a given time/width of the wound at t = 0)] × 100%.

### Transwell assay

The cell migration capacity was measured using modified two-chamber plates as previously described. For the migration assay, MCF-7 and T47D cells were transfected with 50 µM TRIM3 siRNA or siControl. To stimulate migration, the bottom wells were filled with complete medium, while FBS-free medium was added to the upper chambers. After 12 h, the cells were carefully removed, and the cells that invaded through the membrane were fixed and stained with crystal violet staining solution. Cells were counted by using a microscope.

### Lentivirus-mediated knockdown of TRIM3 expression

The lentiviral shTRIM3 vectors were generated via ligation of hybridized oligos (below) into the pLVX lentiviral vector (linearized with BsmBI (NEB)) using T4 DNA ligase (NEB). The sense strand of the nucleotide sequence of the shRNA targeting TRIM3 was 5-CAAACGAAAGGACAACCCA-3. The website of https://www.sigmaaldrich.cn/ was used to design the sequence of shTRIM3. The lentiviral vectors were cotransfected with the psPAX2 and pMD2.G into HEK293T cells using Lipofectamine 2000 (Invitrogen). Medium containing viral particles was collected 48 h after transfection and passed through a 0.45 μM filter. MCF-7 cells were transduced with viral supernatant supplemented with 8 μg/mL polybrene (Sigma–Aldrich). Stably transfected cells were selected with 6 μg/mL puromycin (Solarbio).

### Mouse xenograft model

M-NSG(NOD-Prkd^cscid^IL2rg^em1^/Smoc) mice for the xenograft model were purchased from Shanghai Model Organisms Center, Inc. Four-week-old female M-NSG mice (n = 6 for each group) were implanted with slow-release 17 beta-estradiol pellets (0.72 mg/90-day, Innovative Research of America). After 24 h, approximately 4 × 10^6^ MCF-7 cells together with Matrigel solution were injected into the mammary fat pad of each mouse. The tumor sizes were measured every 5 days. The tumor volume was calculated using the equation volume = (width^2^) × length/2. All animal experiments were approved by the Ethics Committee of Xinxiang Medical University. All animals were kept under a specific pathogen-free (SPF) and temperature-controlled environment with a 12 h light/12 h dark cycle and free access to food and water.

### Western blotting

Cells were harvested and lysed with RIPA buffer. Proteins were separated by SDS–polyacrylamide gel electrophoresis (PAGE) and electrotransferred to PVDF membranes. The antibodies used in this study were as follows: anti-TRIM3 (HAP043396, Sigma, 1:2000), anti-ER alpha (D8H8, 8644, Cell Signaling Technology, 1:2000), anti-HA (MMS-101R, COVANCE, 1:1000), anti-Myc (9E10, ab32, Abcam, 1:1000), anti-actin (A5441, Sigma, 1:10,000), anti-Flag (20543-1-AP, Proteintech, 1:2000), and anti-GFP (ab290, Abcam, 1:4000). Membranes were then washed with PBS three times and incubated with the following secondary antibodies: Peroxidase-Conjugated AffiniPure Goat Anti-Mouse IgG or Goat Anti-Rabbit IgG. Fluorescence signals were visualized with an ECL system (Amersham imager 600, USA).

### Luciferase assay

The luciferase activity of estrogen signaling was determined using the Dual-Luciferase Reporter kit (Promega, Germany). The ERE luciferase reporter was transfected together with the Renilla plasmid into the cells. Luciferase activity was measured after 24 h.

### Coimmunoprecipitation (Co-IP) assay

Immunoprecipitation was performed as described in a previous study [20]. MCF-7 total cell lysates were precleared with rabbit IgG for 2 h and subsequently immunoprecipitated with an anti-ER alpha antibody (SC8005, Santa Cruz, 1:200) overnight, while rabbit IgG (Santa Cruz, 1:200) was used as the negative control. The bound proteins were analyzed by immunoblotting with an anti-TRIM3 antibody (HAP043396, Sigma, 1:2000). For the overexpression experiment, HEK293 cells were transfected with 5 μg of GFP-TRIM3 (full-length or domain deletion mutants) and ER alpha plasmid (full-length or domain deletion mutants) in 10 cm dishes. Cell lysates were precleared with IgG and subsequently incubated with an anti-GFP (ab290, Abcam, 1:200) antibody, while rabbit IgG was used as the negative control. The bound proteins were analyzed by western blotting.

### Polyubiquitination assay

To directly detect the enriched overall ubiquitinated, K63-ubiquitinated, monoubiquitinated and K48-ubiquitinated ER alpha from the cell extracts, HEK293 cells were transfected with 4 μg of Ub, 4 μg of K63 Ubi, 4 μg of Ub-KO or 4 μg of K48 Ubi plasmid, 2 μg of ER alpha, and 0.5 μg of GFP-TRIM3 or GFP-vector. After 48 h, total protein was extracted and precleared with 20 μl protein A (Santa Cruz, SC-2001) for 2 h. The supernatant was collected and immunoprecipitated with an anti-ER alpha antibody (SC8005, Santa Cruz, 1:200) overnight. Western blotting with an anti-HA antibody was performed to detect specific ubiquitinated forms of ER alpha.

### Immunofluorescence assay

MCF-7 cells were fixed with 4% paraformaldehyde in PBS for 10 min, permeabilized with 0.2% Triton X-100 for 5 min and blocked with 5% BSA in PBS for 1 h. Rabbit anti-TRIM3 (HAP043396, Sigma, 1:200) and mouse anti-ERα monoclonal antibodies (SC-56833, 1:200) were used, followed by an Alexa Fluor 647 (Invitrogen, 1:400) anti-rabbit antibody and a FITC-conjugated anti-mouse antibody (Jackson ImmunoResearch, West Grove, PA, 1:400). As negative controls, samples were incubated with secondary antibodies without primary antibodies. Images were acquired under conditions fulfilling the Nyquist criterion using a Nikon A+ laser scanning confocal system with a 60X oil NA1.4 objective and pinhole size of 1.0 Airy units. The acquired images were further processed and assembled using ImageJ.

### Clinical breast tumor samples

One hundred and twenty-three formalin-fixed paraffin-embedded breast cancer samples were collected from the Department of Pathology, Shandong Qilu Hospital. All breast tumor samples were examined to determine the TRIM3 status, ER alpha status, PR status, and HER2 status by pathological specialists. The pathological grade and the lymph node metastasis status of each sample were also examined by pathological specialists. This study was reviewed and approved by the Ethical Board at Qilu Hospital of Shandong University with written informed consent from all patients.

### Analysis of publicly available clinical data

TRIM3 tumor RNA-seq data (TCGA) in breast cancer can be downloaded from the Genomic Data Commons (GDC) data portal website (https://portal.gdc.cancer.gov/). Expression analysis of luminal A-, luminal B-, HER2-positive and triple-negative breast cancer tissues and normal tissues was performed by GraphPad Prism 8. For the correlation analysis between TRIM3 and ER target genes, MORPHEUS (https://software.broadinstitute.org/morpheus/) was used to generate a gene correlation heatmap, and Spearman correlation analysis between genes was performed by cBioPortal (http://www.cbioportal.org/) using 1084 samples of breast invasive carcinoma (TCGA, PanCancer Atlas). Analysis of the association of TRIM3 expression with clinical prognosis was carried out using the KMPLOT database (https://kmplot.com). Analysis of the correlation of TRIM3 expression with ER alpha target gene (TFF1 and GREB1) expression was carried out with 1080 breast cancer samples from the TCGA database.

### Computational analysis of RNA sequencing data

Pathway analysis of differentially expressed genes (DEGs) (*P* value < 0.05 and fold change > 1.5) was performed using hallmark gene sets and KEGG pathways in Metascape (https://metascape.org), and a heatmap was generated by http://www.bioinformatics.com.cn, a free online platform for data analysis and visualization. A volcano plot of the DEGs (threshold *P* < 0.05 and fold change > 1.5) was generated using OmicStudio tools (https://www.omicstudio.cn/tool). For gene set enrichment analysis (GSEA), the HALLMARKS_ESTROGEN_RESPONSE_LATE gene sets were used and downloaded from Molecular Signatures Database v7.4. GSEA was implemented using GSEA 4.1.0 software with default parameters.

### Statistics

Student's t test, Pearson correlation analysis, and Cox regression analysis were used for comparisons. A *P* value of < 0.05 was considered to be significant.

## Results

### TRIM3 expression is elevated in breast tumors and is required for the growth and migration of ER-positive breast cancer

**Fig. 1:**
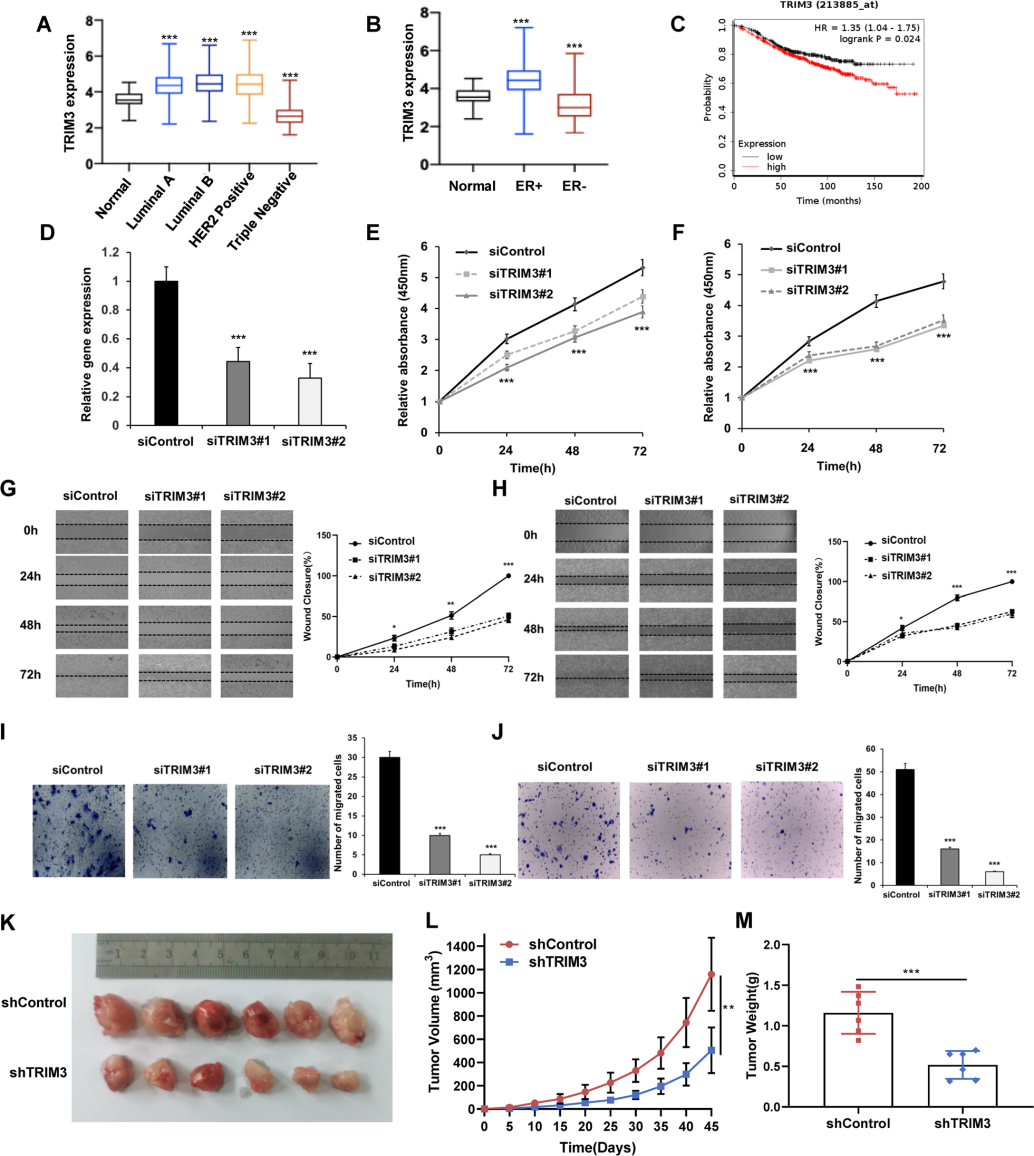
TRIM3 is required for ER alpha-positive breast cancer progression. **a** The distribution of TRIM3 expression in luminal A-, luminal B-, HER2-positive and triple-negative breast cancer tissues and normal tissues. ***P < 0.001. **b** The distribution of TRIM3 expression in ER-positive and ER-negative breast cancer tissues and normal tissues. ***P < 0.001. **c** TRIM3 correlated with poor endocrine therapy outcomes in a publicly available database (https://kmplot.com). **d** TRIM3 mRNA levels in MCF-7 cells were determined by RT–PCR after treatment with TRIM3 siRNA. ***P < 0.001 (Student’s t test). **e** and **f** The CCK-8 assay was used to determine cellular viability at the indicated time points in MCF-7 and T47D cells transfected with siControl or siTRIM3. Experiments were done in triplicate. ***P < 0.001 for cell growth comparisons. **g** and **h** Wound healing assays were utilized to assess the migratory ability of MCF-7 and T47D cells. Quantification of wound closure at the indicated time points. Data are presented as ± SD values. *P < 0.05, **P < 0.01, ***P < 0.001 (Student’s t test). **i** and **j** Transwell assays were utilized to assess the migratory ability of MCF-7 and T47D cells. The cell number was counted, and the data are presented as ± SD values. ***P < 0.001 (Student’s t test). **k**–**m** MCF-7 cells stably transfected with lentivirus carrying shControl or shTRIM3 were subcutaneously injected into the mammary fat pad of each M-NSG mouse. Image of tumors from M-NSG mice transfected with shControl or shTRIM3 MCF-7 cells (**k**). Tumor growth (**l**) and tumor weight (**m**) of subcutaneous xenografts differed significantly in mice injected with shControl and shTRIM3 MCF-7 cells (n = 6; Student's t test; **P < 0.01, ***P < 0.001, shTRIM3 vs. shControl)

In the TCGA database, we analyzed TRIM3 expression in each subtype of breast cancer and found that TRIM3 expression was increased in Luminal A, Luminal B and HER2 positive subtypes compared with normal breast tissues. However, in triple-negative breast cancer, the expression of TRIM3 is opposite (Fig. 1a). In addition, the expression of TRIM3 was significantly elevated in ER-positive breast cancer tissues compared with normal tissues and decreased in ER-negative breast cancer tissues (Fig. 1b). Furthermore, from the survival data analysis, we observed that the high expression of TRIM3 was correlated with a poor survival rate in patients receiving endocrine therapy (Fig. 1c) (https://kmplot.com/analysis/). We utilized two independent siRNAs to test the effect of TRIM3 on breast cancer cells. The knockdown efficiency of the siRNAs is shown in Fig. 1d. In the CCK-8 assay, TRIM3 knockdown decreased the proliferation rate of MCF-7 and T47D cells (Fig. 1e, f). We further investigated the role of TRIM3 in cell migration. The wound healing assay showed that TRIM3 depletion reduced the wound closure rate in both MCF-7 and T47D cells (Fig. 1g, h). The trans-well assay indicated that TRIM3 depletion reduced the cell migration capability in both MCF-7 and T47D cells (Fig. 1i, j). Next, we investigated the role of TRIM3 in tumor growth in xenograft mouse models. Our data showed that TRIM3 depletion by lentivirus-based shRNA slowed breast tumor growth (Fig. 1k–m). All these experiments might indicate the oncogenic role of TRIM3 in ER-positive breast cancer cells.

### TRIM3 depletion decreases the expression of ER target genes in breast cancer cells

**Fig. 2:**
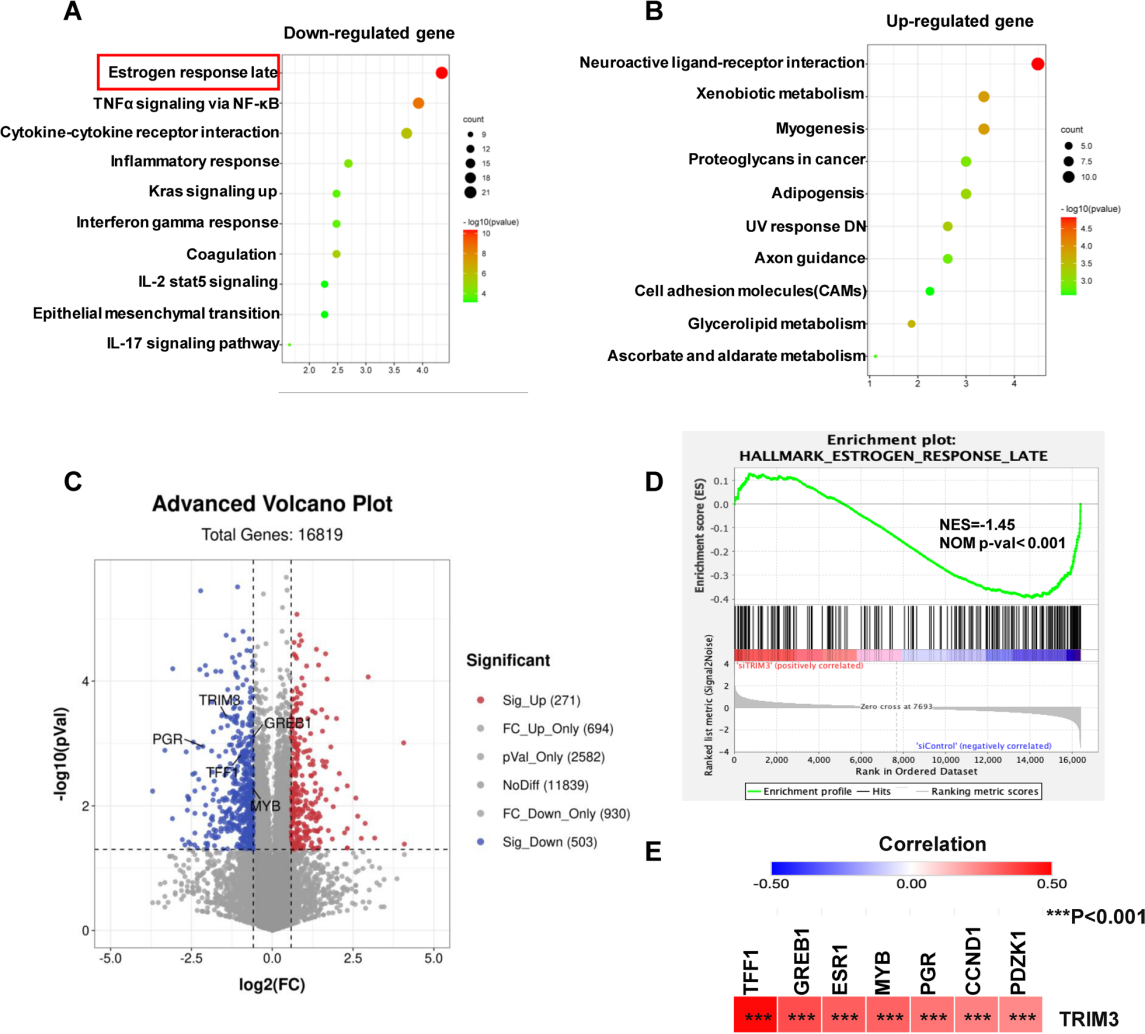
TRIM3 modulates estrogen signaling in breast cancer cells. **a** and **b** KEGG analysis of downregulated (left) and upregulated (right) genes in RNA-seq data of TRIM3 siRNA MCF-7 cells. **c** Volcano plot shows that canonical ER target genes are enriched in downregulated genes in TRIM3 siRNA MCF-7 cells. Threshold *P* < 0.01 and fold change > 2. **d** Gene set enrichment analysis (GSEA) shows enrichment of estrogen response genes in TRIM3 siRNA MCF-7 cells. **e** Heatmap of the correlation between TRIM3 and ER signaling target genes in breast invasive carcinoma (TCGA, PanCancer Atlas). Different colors represent correlation coefficients (in the diagram, red represents a positive correlation, blue represents a negative correlation), and darker colors represent stronger correlations. Asterisks represent levels of significance (***P < 0.001)

Since we showed that TRIM3 is an important factor in cell proliferation and migration in ER-positive breast cancer cells, we further investigated the role of TRIM3 at the transcriptome-wide scale. We depleted TRIM3 in MCF-7 cells for whole-genome expression analysis. Hallmark gene set and KEGG pathway analysis in Metascape (https://metascape.org) showed that TRIM3 depletion affects several aspects of cancer biological processes. Interestingly, among the downregulated signaling pathways, the estrogen signaling pathway ranked first (Fig. 2a, b). The volcano plot showed that canonical ER target genes were enriched among the downregulated genes in TRIM3 siRNA MCF-7 cells (Fig. 2c). GSEA in Molecular Signatures Database v7.4 showed that the “estrogen response” gene set was enriched in breast cancer cells, suggesting a regulatory effect of TRIM3 on cancer cells in response to ER signaling (Fig. 2d). The heatmap showed that TRIM3 depletion affected a group of ER alpha signature genes, for example, TFF1 and GREB1 (Fig. 2e).

### The TRIM3 level correlates with the ER alpha protein level and the mRNA levels of its target genes in breast cancer

**Fig. 3:**
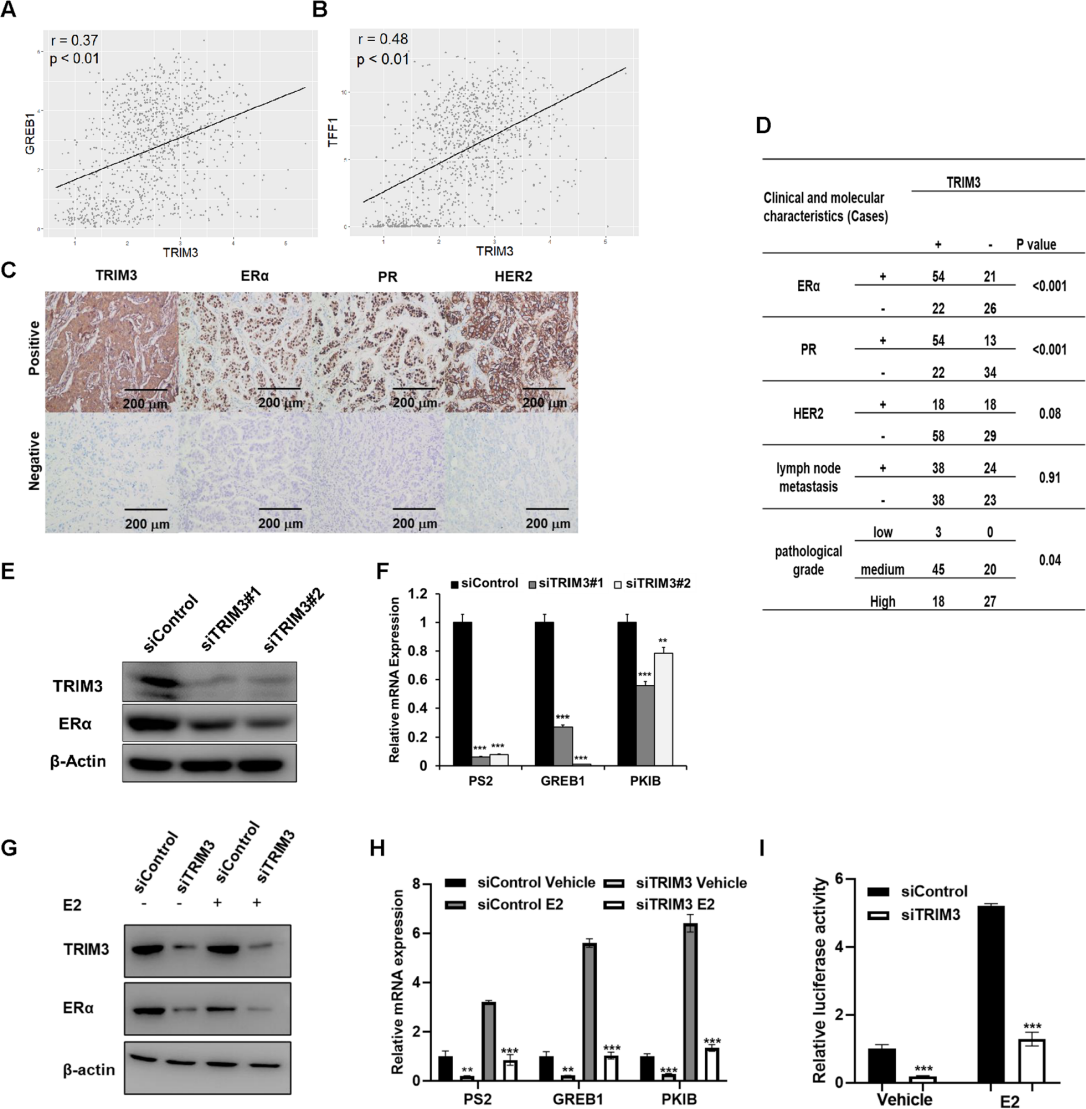
TRIM3 correlates with the ER alpha protein level and mRNA levels of its target genes in breast cancer samples. **a** and **b** Publicly available data show that TRIM3 was positively correlated with the ER alpha target genes GREB1 and TFF1 (https://www.cbioportal.org). **c** Example tumor cases showing that TRIM3 protein expression is positively correlated with ER alpha and PR protein expression in IHC analysis. **d** Statistical analysis of the correlation of TRIM3 with ER alpha expression in 123 human breast tumor samples. **e** TRIM3 and ER alpha protein levels were determined by western blotting. Actin was used as internal control. **f** PS2, GREB1, and PKIB mRNA levels were determined by RT–PCR after treatment with TRIM3 siRNA in MCF-7 cells. ***P < 0.001 for target gene expression comparison. **g**–**i** MCF-7 cells in charcoal-stripped FBS and phenol red-free DMEM were transfected with siTRIM3 or siControl. After 48 h, cells were treated with either ethanol or 10 nM estradiol for 6 h. TRIM3 and ER alpha protein levels were determined by western blot analysis. Actin was used as internal control (**g**). PS2, GREB1, and PKIB mRNA levels were determined by RT–PCR (**h**). Luciferase activity was measured after transfection (**i**). Data are presented as ± SD values. ***P < 0.001 (Student’s t test)

We further investigated the relationship between the expression of TRIM3 and canonical ER alpha target genes. In the TCGA database, we observed that TRIM3 expression was correlated with TFF1/GREB1 expression in 1080 breast tumor samples (Fig. 3a, b) (https://tcga-data.nci.nih.gov/tcga/). To analyze the correlation of the TRIM3 expression level and the expression levels of breast cancer molecular biomarkers, 123 invasive ductal breast cancer tissues were collected for immunohistochemical (IHC) analysis. We examined the protein expression levels of TRIM3, ER alpha, PR and HER2 (Fig. 3c). In addition, clinicopathological features, including pathological grade and lymph node metastasis status, were also collected. IHC analysis indicated that the protein level of TRIM3 was correlated with the ER alpha protein level (*P* < 0.001) and the PR protein level (*P* < 0.001). In addition, the TRIM3 expression level was correlated with higher pathological grade (*P* = 0.04) (Fig. 3d). Further RT–PCR experiments showed that TRIM3 depletion reduced the expression of canonical ER alpha target genes, such as PS2 and GREB1 (Fig. 3e, f). We further tested the effect of TRIM3 on ER alpha signaling activity in both vehicle-treated and E2-treated conditions. TRIM3 depletion reduced the ER alpha protein level and ER alpha target gene expression levels in vehicle-treated and E2-treated MCF-7 cells (Fig. 3g, h). To determine whether TRIM3 knockdown can influence ER alpha transcriptional activity, we performed an estrogen response element (ERE) luciferase assay in MCF-7 cells, which showed that TRIM3 depletion decreased ERE luciferase activity in MCF-7 cells (Fig. 3i).

### TRIM3 associates with ER alpha and modulates ER alpha protein stability

**Fig. 4:**
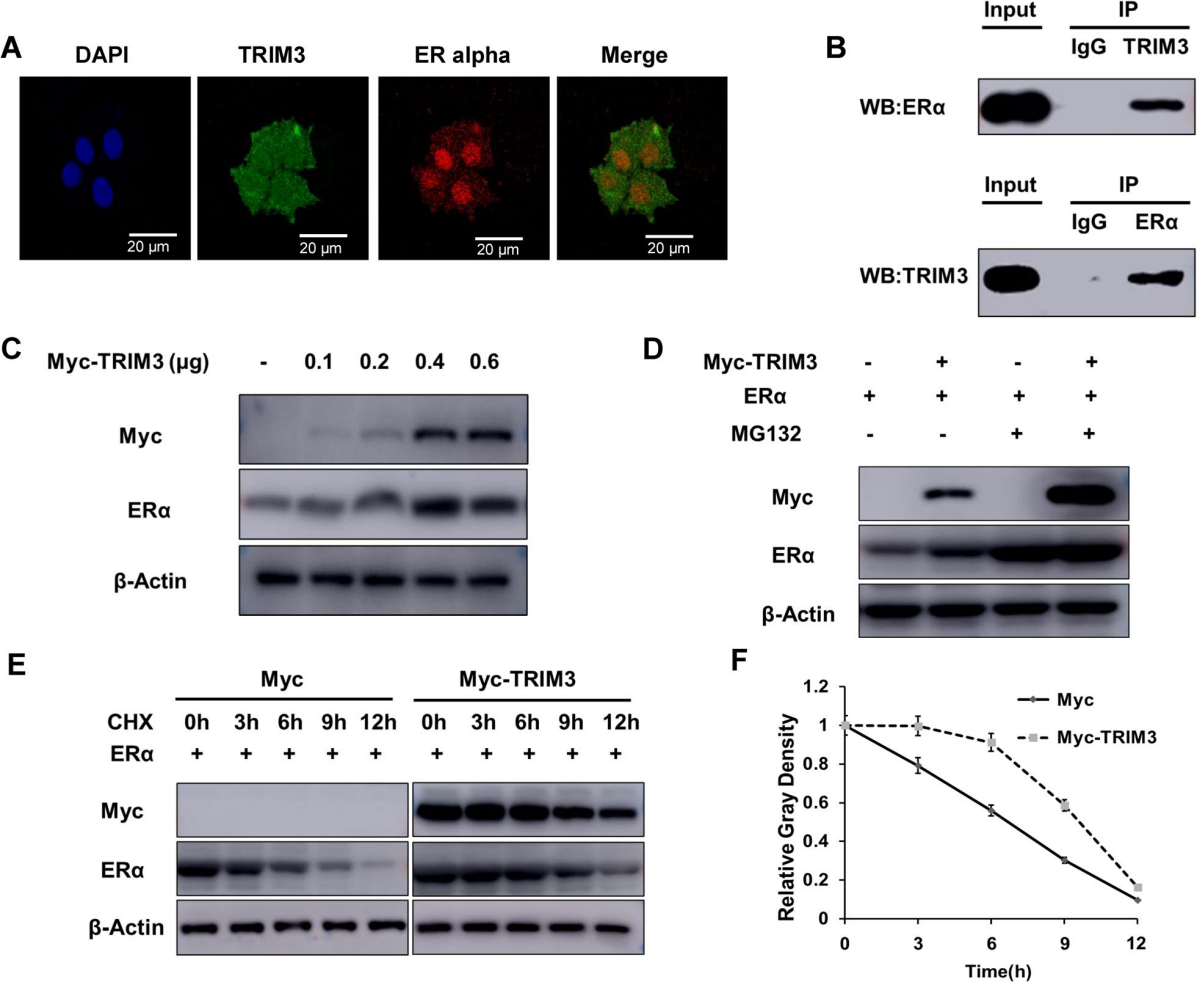
TRIM3 associates with ER alpha and modulates ER alpha stability. **a** Intracellular localization of ER alpha (red) and TRIM3 (green) is shown. Nuclei (blue) were stained with 4′,6-diamidino-2-phenylindole (DAPI) in MCF-7 cells. **b** Co-IP was performed using antibody as indicated. **c** ER alpha protein levels were determined by western blotting. HEK293 cells were transfected with 1 μg ER alpha together with different amounts of TRIM3 plasmids. **d** ER alpha protein levels were determined by western blotting. Actin was used as internal control. HEK293 cells were transfected with TRIM3 and ER alpha plasmids. After 24 h, cells were treated with 10 μM MG132 or vehicle for 6 h. **e** and **f** ER alpha protein levels were determined by western blotting. Actin was used as internal control. HEK293 cells were transfected with HA-ERα plasmid and Myc-tagged or Myc-TRIM3 plasmids. After 24 h, cells were treated with 100 µM cycloheximide (CHX) for the indicated times. The results are representative of three independent experiments. The relative density of ER alpha was measured by ImageJ software

We further examined the protein localizations of TRIM3 and ER alpha in breast cancer cells. Immunofluorescence staining indicated that ER alpha was mainly localized in the nucleus, while TRIM3 was localized in both the cytosol and the nucleus (Fig. 4a). The endogenous immunoprecipitation (IP) assay showed that the TRIM3 protein can associate with the ER alpha protein in MCF-7 cells (Fig. 4b). We further investigated the biological function of this association. ER alpha can promote its own mRNA transcription, making it difficult to distinguish the direct role of TRIM3 in modulating ER alpha mRNA or protein levels in the cell lines with endogenous ER alpha expression. We utilized HEK293 cells, which do not exhibit endogenous ER alpha expression, to investigate the mechanism. Co-overexpression of ER alpha and TRIM3 in HEK293 cells showed that TRIM3 elevated the ER alpha protein level (Fig. 4c), an effect that was minimized by treatment with the proteasome inhibitor MG132 (Fig. 4d). The protein half-life assay showed that TRIM3 increased the protein stability of ER alpha (Fig. 4e, f).

### TRIM3 interacts with the ER alpha DNA binding domain via its filamin/NHL domain

**Fig. 5:**
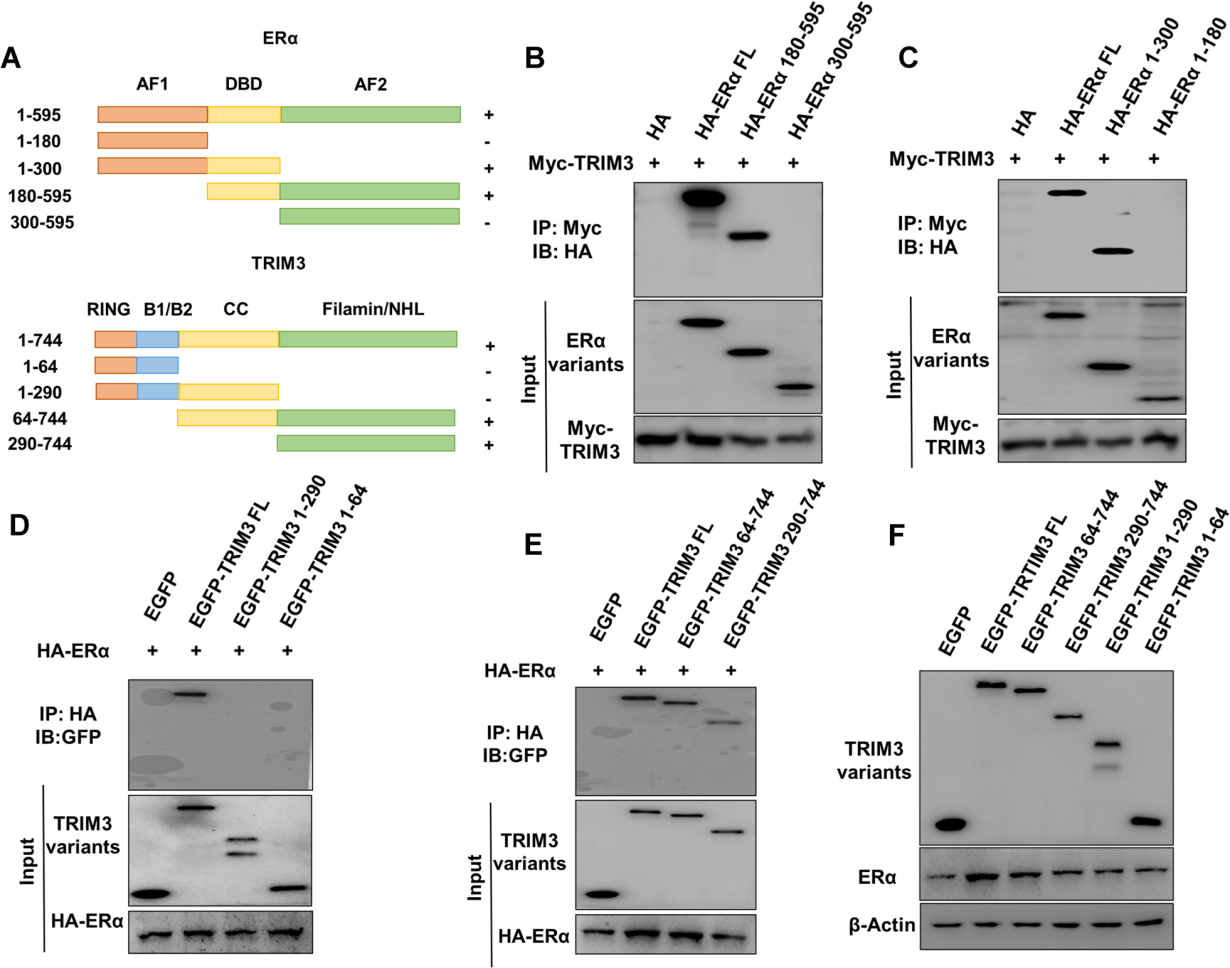
TRIM3 associates with the ER alpha DNA binding domain via its filamin/NHL domain. **a** ER alpha and TRIM3 domain structures used in this study. **b** and **c** The possible interacting domains in ER alpha were detected by an anti-HA antibody. HEK293 cells were transfected with 2 µg of Myc-TRIM3 together with HA-ER alpha full length or mutants (AF1, AF1 + DBD, AF2 and AF2 + DBD). Co-IP was performed using an anti-Myc antibody. **d** and **e** The possible interacting TRIM3 domains were detected by GFP antibody. HEK293 cells were transfected with 2 µg of HA-ER alpha together with GFP-TRIM3 full length or mutants (RING + B1/B2, RING + B1/B2 + CC, filamin/NHL and filamin/NHL + CC). Co-IP was performed using an anti-HA antibody. **f** ER alpha protein levels were detected via western blotting. HEK293 cells were transfected with 2 µg of Flag-ER alpha and 0.5 µg of GFP-TRIM3 full length or mutants (RING + B1/B2, RING + B1/B2 + CC, filamin/NHL and filamin/NHL + CC)

We further characterized the association domains between ER alpha and TRIM3. ER alpha consists of three main functional domains: a DNA binding domain (DBD) and two domains with a transcriptional activation function (AF1 domain and AF2 domain), while TRIM3 contains a RING domain, B1/B2 domain, CC domain and filamin/NHL domain. We constructed the ER alpha variants AF1 (1–180 aa), AF1 + DBD (1–300 aa), AF2 (300–595 aa) and AF2 + DBD (180–595 aa) and the TRIM3 variants RING + B1/B2 (1–64 aa), RING + B1/B2 + CC (1–290 aa), filamin/NHL (290–744 aa) and filamin/NHL + CC (64–744 aa) (Fig. 5a). The IP results indicated that the DBD domain of ER alpha is required for its association with TRIM3 (Fig. 5b, c), while the filamin/NHL domain of TRIM3 is needed for its association with ER alpha (Fig. 5d, e). However, co-overexpression of ER alpha with the TRIM3 variants showed that only full-length TRIM3 stabilized the ER alpha protein (Fig. 5f).

### TRIM3 facilitates K63-linked ubiquitination and mono-ubiquitination of ER alpha

**Fig. 6:**
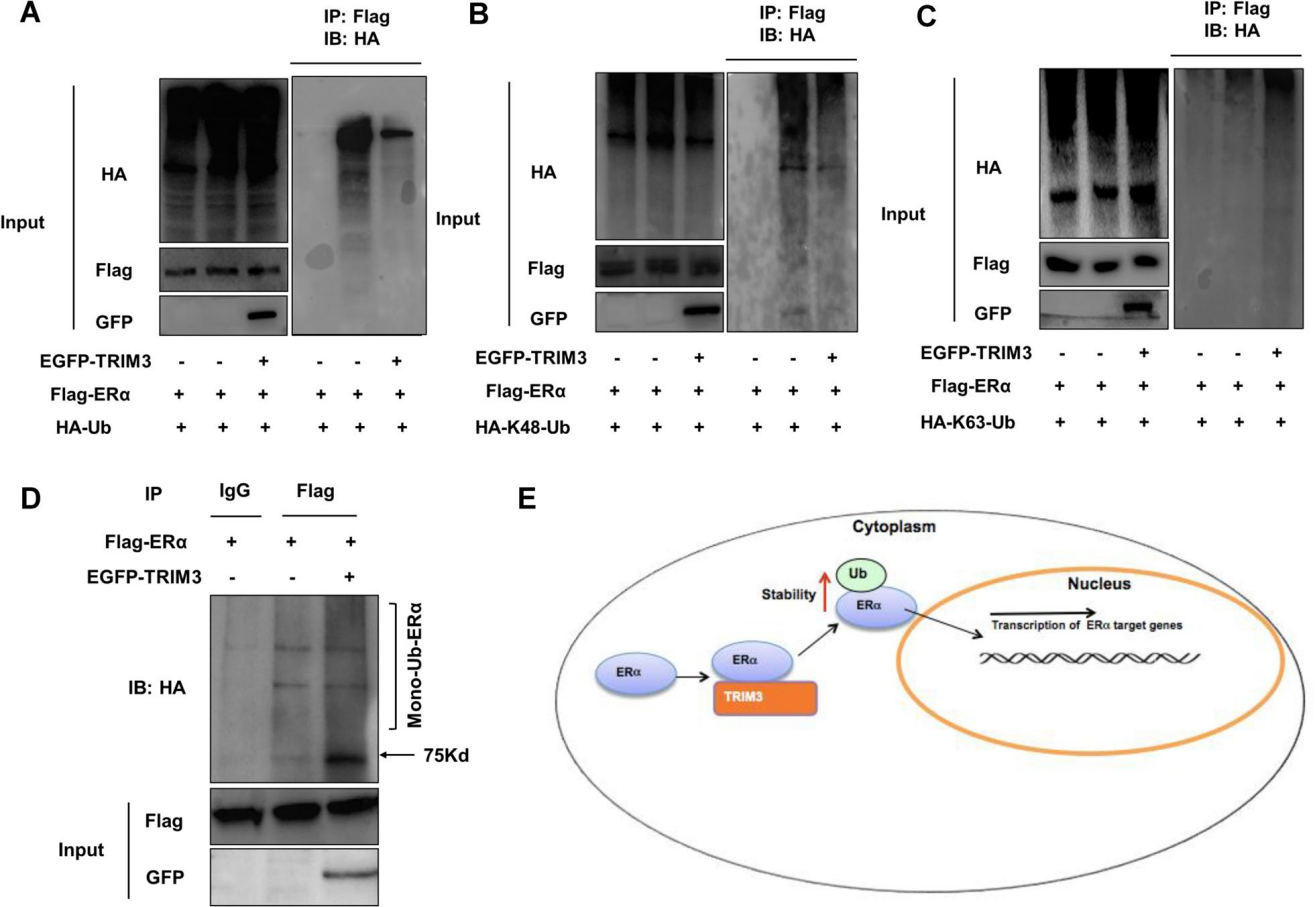
TRIM3 facilitates K63-linked ubiquitination and monoubiquitination of ER alpha. **a** Polyubiquitinated ER alpha was detected via western blotting. **b** K48-specific polyubiquitinated ER alpha was detected via western blotting. **c** K63-specific polyubiquitinated ER alpha was detected via western blotting. **d** Monoubiquitinated ER alpha was detected via western blotting. **e** The hypothetical model of the mechanism by which TRIM3 regulates ER alpha signaling in breast cancer: TRIM3 interacts with the ER alpha protein, stabilizes ER alpha and facilitates its signaling activation and breast cancer progression

Since TRIM3 is a putative E3 ubiquitin ligase, we further investigated the role of TRIM3 in ER alpha ubiquitination. Ubiquitination-based IP showed that TRIM3 inhibited ER alpha protein polyubiquitination (Fig. 6a). Among the ubiquitination mechanisms, K48-linked ubiquitination is the most common degradation mechanism. We examined the role of TRIM3 in ER alpha K48-linked ubiquitination, which implied that TRIM3 could suppress K48-linked ubiquitination of ER alpha (Fig. 6b). In addition, since K63-linked ubiquitination or mono-ubiquitination of ER alpha can increase ER alpha stability and signaling activity, we further analyzed the effect of TRIM3 on these two types of ER alpha ubiquitination. The data indicated that TRIM3 could significantly promote K63-linked polyubiquitination and mono-ubiquitination of the ER alpha protein (Fig. 6c, d).

## Discussion

In our current study, we identified one RING family protein, TRIM3, that modulates ER alpha signaling via increasing its stability (Fig. 6e). Analysis of clinical data indicated a positive correlation between TRIM3 and ER alpha protein levels in human IHC samples. In addition, TRIM3 correlated with poor survival in endocrine therapy patients. Our study identifies a nongenomic regulatory mechanism of ER alpha stability control. Based on these findings, we propose that modulation or inhibition of the TRIM3 protein could be an interesting strategy for ER alpha-positive breast cancer treatment.

The ER alpha gene was first discovered 30 years ago [7]. Based on the current understanding of breast cancer, ER alpha signaling is the main driver for two-thirds of breast cancer patients [22]. Although gene amplification or mutation of ER alpha is not common in breast cancer, increased ER alpha expression can be observed in most breast tumors compared with normal breast tissue [23]. Thus, targeting ER alpha signaling is an excellent strategy for breast cancer, while the selective modulator of ER alpha, including tamoxifen, demonstrated great success in inhibiting ER alpha signaling and breast cancer progression [22]. However, the occurrence of endocrine resistance is a challenging issue in breast cancer therapy. Interestingly, most endocrine-resistant breast tumors still maintain ER alpha protein expression, which lends to the possible involvement of ER alpha in drug resistance. Several confirmed and possible mechanisms indicate that the crosstalk between ER alpha and other signaling pathways or ER alpha modifications plays important roles in medicating endocrine resistance. For example, ER alpha can transactivate several growth factor pathways, including IGFR and AKT signaling, while the activation of these signaling cascades promotes the phosphorylation of ER alpha and facilitates ER alpha transactivation [24]. In addition, recent studies have revealed that protein modifications of ER alpha can stabilize ER alpha or enhance its transcriptional activity in breast tumors [10, 25]. Thus, modulating the ER alpha expression level and protein stability could be an effective strategy for overcoming endocrine resistance.

There are approximately 500–1000 E3 ubiquitin ligases, which can be divided into four groups: HECT type, RING type, U-box type and PHD-finger type [26]. Among these, the RING family is the largest, and its function is not totally clear. Interestingly, recent studies have shown that some RING family E3 ligases prefer to catalyze in an atypical ubiquitin manner, which does not necessarily lead to degradation [27]. Several RING family members participate in ER alpha signaling activity and breast cancer progression via non-degradation related ubiquitination. For example, RNF8 was shown to associate with ER alpha, promoting increased ER alpha monoubiquitination and signal transduction [28]. In addition, our previous work identified a few E3 ubiquitin ligases that modulate ER alpha stability in a nongenomic manner, including TRIM56 and RNF31 [29, 30].

TRIM3 belongs to the RING finger family of proteins and is composed of a zinc-binding domain, RING domain, B1/2 domain and coiled-coil region [19]. TRIM3 was first reported to interact with myosin and promote the transport of target proteins [15]. TRIM3 expression is decreased in several human cancers, including gastric cancer, colon cancer and liver cancer [18, 19, 31, 32]. However, the reports regarding TRIM3 in breast cancer are not consistent. Yongzhen Li et al. reported that TRIM3 was a tumor suppressor in breast cancer and related to longer overall survival of BC patients [33]. Yet, another two studies reported the TRIM3 played oncogenic roles in breast cancer. The regulatory mechanisms of TRIM3 are mainly due to the post-translational suppression of P53 protein function and sumoylation on estrogen receptor, which facilitate estrogen signaling and breast cancer proliferation [34, 35]. Our current study reveals similar conclusions with these two studies, in which TRIM3 is elevated in luminal type of breast cancer and promotes breast cancer progression. These findings further validate the conclusion that TRIM3 mainly functions as an oncogene in breast cancer. However, our molecular assays reveal a different regulatory mechanism. In our study, TRIM3 can facilitate ER alpha signaling, promoting cell proliferation and migration in breast cancer via enhancing ER alpha stability possibly via promoting ER alpha K63-linked poly-ubiquitination. This interesting finding not only increases the understanding of ER alpha posttranslational modifications but also implies the multiple functions of TRIM3 in different cancer backgrounds.

## Conclusions

We identified an interesting E3 ligase, TRIM3, that facilitates ER alpha signaling in breast cancer cells. TRIM3 can promote breast cancer cell migration and proliferation by stabilizing the ER alpha protein. As TRIM3 is a novel modulator of ER alpha signaling, disrupting its protein expression or activity could be a plausible strategy to treat ER alpha-positive breast cancer.

## Data Availability

The RNA sequence data are deposited in the Gene Expression Omnibus (GEO) database (accession number: GSE143610).

## Abbreviations

TRIM3: Tripartite motif containing 3
ER alpha (ERα): Estrogen receptor alpha
AF1: Transcriptional activation domain 1
DBD: DNA binding domain
AF2: Transcriptional activation domain 2
HER2: Human epidermal growth factor receptor 2
PR: Progesterone receptor
ATCC: American type culture collection
